# Comparative study on numerical simulation of temperature field of farm house with different roof forms

**DOI:** 10.1038/s41598-024-54751-0

**Published:** 2024-04-02

**Authors:** Zhichao Pan, Wenjuan Zhao, Haidong Wang

**Affiliations:** 1 School of Mathematics and Information Science, North Minzu University, Mathematics, Yinchuan, 750021 Ningxia China; 2 School of Architecture Ningxia University, Ningxia University, Yinchuan, 750021 Ningxia China; 3 College of Water Conservancy & Hydropower Engineering, Hohai University, Nanjing, 210098 Jiangsu China

**Keywords:** Engineering applications, Model of thermal mass transport, Periodic initial boundary conditions, Numerical simulation, Architectural structure, Engineering, Civil engineering, Engineering, Civil engineering

## Abstract

In recent years, the issue of energy consumption in farm buildings has received much attention. The roofs of farm buildings in Northwest China have a variety of roof forms. This paper presents the implementation of first fully confirmed the indoor thermal environment of different roof construction was significantly effected by periodic thermogenesis. In order to determine the indoor temperature distribution of the farmhouse in summer in Ningxia Hui Autonomous Region, we provided the heat transfer coefficient data of the farmhouse envelope, also detailed in the manuscript. Model of Thermal Mass Transport enables fast and accurately simulates the indoor temperature distribution of farmhouses with different roof forms on the same day, taking into account the climate zone of the region. This is despite the phase delay time of indoor temperatures for different roof forms caused by periodic initial temperature boundaries ranged from 1.55 to 2.78$\text{h}$, and the phase delay angle ranged from 23.25^∘^ to 41.7^∘^. Extensive simulated results revealed individual variability in the role of roof form, demonstrating indoor temperatures in farmhouses corresponding to different climatic zones. In addition, by analyzing and discussing the indoor temperature phase delay angle and delay time for each type of roof forms, statistical results identified the advantages of Non-equal-sloped roof as a local farmhouse roof.

## Introduction

The total amount of rural energy consumption and the structure of energy consumption are undergoing profound changes, and people are putting forward higher requirements for the thermal comfort of farm buildings as the material living standard of rural residents rises^1^. However, the existing pattern of energy use in farm buildings is relatively crude, so problems such as poor thermal insulation, rapid energy dissipation, and inefficient utilization of energy arise^2^.

The roof should have excellent thermal properties in addition to a good load-bearing capacity as part of the top envelope of the building. Because the roof is in a special location, it is exposed to the sun for a long time. It gets the most heat, which results in a large amount of heat going in and out of the building through the roof, leading to higher indoor temperatures in the summer and lower indoor temperatures in the winter. Combined with the above reasons, for rural dwellings, improving the thermal performance of roofs can achieve the goal of creating a comfortable indoor thermal environment for farm structures^3^.

Domestic and foreign researchers have carried out a number of studies on the energy-saving retrofit of roof forms in the context of changing concepts of building development. The conclusion that naturally ventilated roofs can effectively improve the thermal environment of factory buildings and reduce energy consumption in factory buildings was reached through a study by L. Susanti L et al, from Toyohashi Institute of Technology, Japan. Specifically, the temperature on the inner surface of a single-ply solid roof is higher than the temperature on the upper surface of an elevated roof between 8 am and 6 pm^4^. Nandapala K et al. proposed a discontinuous support strip insulation system for roof panels at Moratuwa University, Kathubeda, Sri Lanka. They proved through tests that the system was even better than a wood-ceilinged floral tile roof in terms of thermal performance^5^. A study by Salah-Eddine Ouldboukhitine et al. at the University of La Rochelle, France, found that green roofs protect roof membranes from heat fluctuations and then reduce the maximum summer roof surface temperature by 20 degrees Celsius, thereby increasing roof life. It also delays the peak temperature of the membrane surface by a few hours^6^. Reihaneh Aghamolaei et al. at the University of Tehran have made progress in improving OTC modeling of building roof areas. They utilized computational fluid dynamics (CFD) and building energy simulation (BES) methods to develop a high-resolution, but computationally efficient OTC simulation framework to couple radiant and convective fluxes in outdoor environments and also invoked PET coefficients as an OTC metric to reflect human thermal comfort^7^.

Qiman Hu of Chongqing University analyzed the impact of the thermal performance of the roof envelope on the indoor thermal environment of rural dwellings. A thermal insulation and cooling program to improve the indoor thermal environment using a combination of roof insulation measures and appropriate ventilation was proposed by her^8^. Xiao Jian from Hunan University introduced the structural design of sloped roof buildings and analyzed the significance of roof renovation in hot summer and cold winter areas^9^.

In the current scientific research results, most of the main discussions among researchers focus on how to improve the thermal insulation and heat preservation capacity of the roof envelope from the direction of building energy efficient roof retrofit^10,11^. However, not much research has been done on the effect of roof form on the energy efficiency of the roofs of farm buildings under climate zoning^12^. The analysis of the distribution of the indoor temperature field of various types of roof forms in different climatic zones can be helpful for energy-saving roof retrofits based on the fact that roofs have a strong effect on the indoor thermal environment of buildings^13,14^.

The purpose of this paper is to carry out a heat transfer analysis study of farm buildings in Ningxia region using ANSYS Fluent software. Through numerical simulation using periodic boundary conditions and accurate comparative analysis of the indoor temperature field distribution of farmhouses under different roof forms from the perspective of mathematical-physical modeling, reference suggestions are provided for the practice of energy-saving retrofitting of farmhouse roofs in this region.

The research on heat transfer through building roofs is currently limited. The current paper aims to investigate the impact of the structural form of the roof of agricultural buildings on the distribution of the indoor temperature field.

This study aims to determine the optimal form of roof structure for different climate zones, considering building energy consumption. The current paper focuses on selecting the most suitable roof structure for each specific local climate zone.

Previous studies on indoor temperature distributions in farmhouses have overlooked the impact of initial periodic boundary conditions when employing unsteady-state calculations. This could be attributed to the fact that previous studies primarily considered the average outdoor temperature as the boundary condition. Consequently, phase delay angles and delay times of indoor and outdoor are significant in comparison. Calculations revealed that the phase delay time of indoor temperatures for different roof forms caused by periodic initial temperature boundaries ranged from 1.55$\text{h}$ to 2.78$\text{h}$, and the phase delay angle ranged from 23.25^∘^ to 41.7^∘^.

## Methods

### Physical model

**Figure 1:**
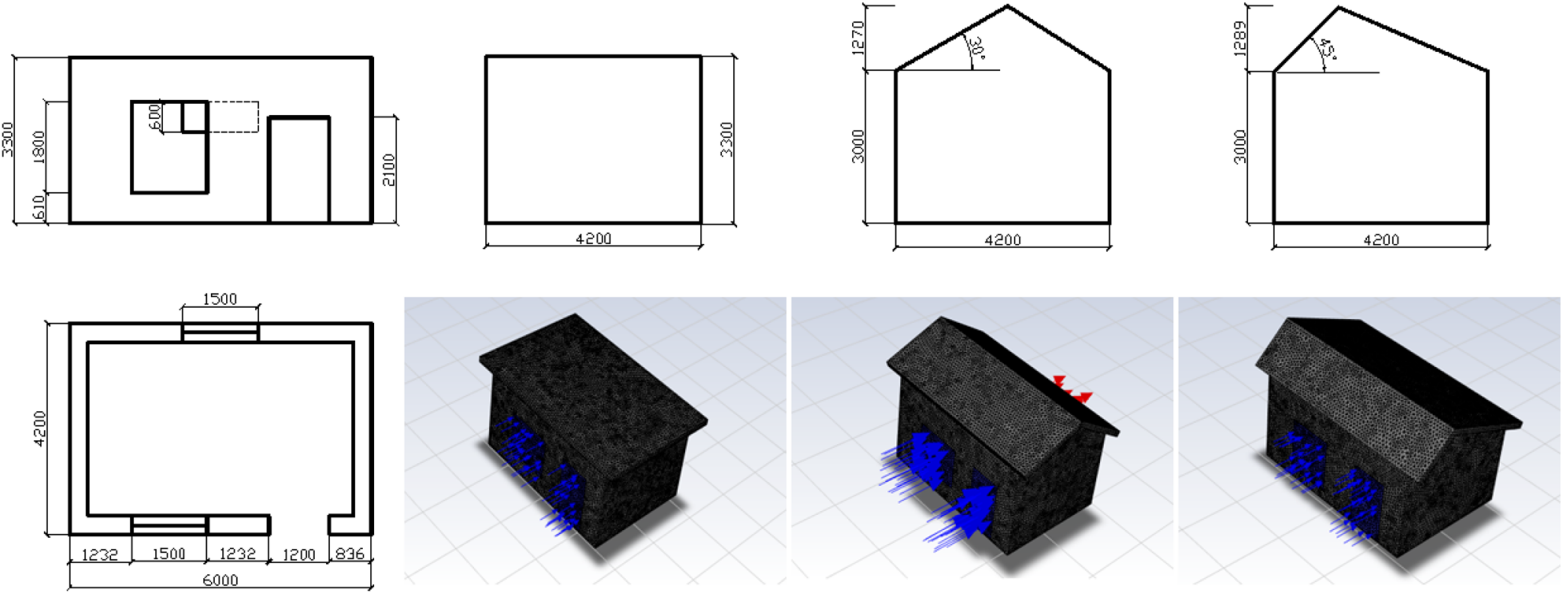
Design drawing and 3D model of experimental house.

The size (length, width and height) of the physical model in this study is $L \times W \times H = 6\text{m} \times 4.2\text{m} \times 3\text{m}$. Local farm buildings in Ningxia mainly have Flat roofs and Double-sloped roofs, of which the Double-sloped roofs are divided into Non-equal-sloped roof structures and Equal-sloped roof structures. At the same time, three 3D model of the experimental house was established using SpaceClaim software. Models were actuated by direction of dominant summer winds in Ningxia, direction represented 15^∘^ 20.26^′^ S.E.. The simulation date was chosen a day of the great summer heat in the Chinese lunar calendar. The design diagram and calculation model can be shown in Fig. 1.

1 $$\begin{aligned} K=\left( 0.15+\sum \frac{d}{\lambda } \right) ^{-1} . \end{aligned}$$

The Hot Disk pt1500 thermal constant analyzer from Sweden was used to test the thermal physical parameters of soil samples, with the testing range of thermal conductivity from 0.005 to 500$\text{W} /\left( \text{m} \cdot \text{K} \right)$ and the accuracy of $\pm 3\%$. the accuracy is $\pm 7\%$ and $\pm 5\%$ for the volume specific heat and thermal diffusion coefficient, the temperature range is from – 26 to 700^∘^C. From actual measurement, and then through the Eq. (1) can be calculated the Heat Transfer Coefficient of Roof, Ground, and External wall. The thermophysical parameters of the experimental house enclosure are presented in the following Table 1.

**Table 1:** Thermophysical parameters of experimental house.

Demonstration enclosure structure	Roof	Ground	External wall 1	External wall 2	External wall 3
*K* $(\text{W} /\left( \mathrm {m^2} \cdot \text{K} \right) )$	0.21	0.3	0.581	0.540	2.015

### Mathematical model of thermal mass transport

$k-\omega$ model proposed by Menter FR (2009) was used to parametrically modify by Fluent software, which performs optimally at building walls and does not require a low Reynolds number correction. At the same time, the $k-\omega$ model is combined with the standard $k-\varepsilon$ model which is less affected by the flow at the boundary layer and outer edges of the fluid domain, and is switched using the appropriate mixing function. In contrast, the *SST* $k-\omega$ model limits vortex viscosity by forcing turbulent shear stress^15^. This improvement improves the performance of the model for strong inverse pressure gradients and separated flows. Additionally, the full formulation of the mathematical model for thermal mass transport is provided, where the turbulent kinetic energy *k* and turbulent frequency $\omega$ can be obtained from the following thermal mass transport equations^16,17^:

2 $$\begin{aligned} \left\{ \begin{array}{l} \frac{\partial (\rho k)}{\partial t}+\frac{\partial \left( \rho u_i k\right) }{\partial x_i} =\frac{\partial }{\partial x_j}\left[ \left( \mu +\sigma _k \mu _t\right) \frac{\partial k}{\partial x_j}\right] +p_k-\rho \omega k \beta ^* \\ \frac{\partial (\rho \omega )}{\partial t}+\frac{\partial \left( \rho u_i \omega \right) }{\partial x_i} =\frac{\partial }{\partial x_j}\left[ \left( \mu +\sigma _k \mu _t\right) \frac{\partial \omega }{\partial x_j}\right] +\frac{\gamma }{v_t} p_k-\beta \rho \omega ^2 +2\left( 1-F_1\right) \frac{\rho \sigma _{\omega 2}}{\omega } \frac{\partial k}{\partial x_j} \frac{\partial \omega }{\partial x_j} \\ v_t=\frac{\rho k a_1}{\max \left( a_1 \omega , S F_2\right) } \\ \frac{\partial (\rho E)}{\partial t}+\frac{\partial }{\partial x_i}\left[ u_i(\rho E+p)\right] =\frac{\partial }{\partial x_i}\left[ \left( k_{eff}+\frac{c_p \mu _t}{pr_t}\right) \frac{\partial T}{\partial x_i} -\sum _j h_j J_j+u_i\left( \tau _{ij}\right) _{eff}\right] +S_h \end{array}\right. \end{aligned}$$

where

3 $$\begin{aligned} \left\{ \begin{array}{l} S=\sqrt{2 S_{i j} S_{i j}}, p_k=\min \left( \mu _t \frac{\partial U_i}{\partial x_j}\left( \frac{\partial U_i}{\partial x_j}+\frac{\partial U_j}{\partial x_i}\right) , 10 \cdot \beta ^* \rho k \omega \right) \\ F_1=\tanh \left\{ \left\{ \min \left[ \max \left( \frac{\sqrt{k}}{\beta ^* \omega y}, \frac{500 v}{y^2 \omega }\right) , \frac{4 \rho \sigma _{\omega 2} k}{C D_{k \omega } y^2}\right] \right\} ^4\right\} \\ F_2=\tanh \left[ \left[ \max \left( \frac{2 \sqrt{k}}{\beta ^* \omega y}, \frac{500 v}{y^2 \omega }\right) \right] ^2\right] \\ C D_{k \omega }=\max \left( 2 \rho \sigma _{\omega 2} \frac{1}{\omega } \frac{\partial k}{\partial x_i} \frac{\partial \omega }{\partial x_i}, 10^{-10}\right) \end{array}\right. \end{aligned}$$

where *y* is the distance closest to the wall; *S* is the strain rate; $\rho$ is the density of air; $p_{k}$ is the pressure. This study focuses on incompressible gases, so $h=\sum _{j^{\prime }} Y_{j^{\prime }} h_{j^{\prime }}+\frac{p_k}{\rho }$. The specific heat capacity is represented by symbol $c_{p}$ ; The thermal conductivity of the fluid is represented by symbol $k_{eff}$. The source term for the conversion of the mechanical energy of the fluid into thermal energy due to viscosity is represented by symbol $S_{h}$. $F_{1}$ is the mixing function that is zero away from the surface ( $k-\varepsilon$ model); $F_{2}$ is the mixing function switching to the interior of the boundary layer ($k- \omega$ model).

It is worth noting that the *SST* $k-\omega$ model of the thermal mass transport model uses a yield limiter to prevent the formation of turbulence in the stagnation zone, which is one of the most important parts of the *SST* $k-\omega$ model^18^. And each of the constants $\phi$(including $\beta$,$\sigma _k$,$\sigma _{\omega 2}$, $\gamma$,etc.) are calculated by mixing the corresponding constants from the $k-\varepsilon$ model and the $k- \omega$ model, is^19,20^:

4 $$\begin{aligned} \phi =F_1 \phi _1+\left( 1-F_1\right) \phi _2. \end{aligned}$$

The constants of the model are shown below:

5 $$\begin{aligned} \left\{ \begin{array}{l} \gamma _1=\frac{\beta _1}{\beta ^*}-\frac{\sigma _{\omega 1} \kappa ^2}{\sqrt{\beta ^*}} \\ \gamma _2=\frac{\beta _2}{\beta ^*}-\frac{\sigma _{\omega 2} \kappa ^2}{\sqrt{\beta ^*}} \\ \sigma _{k 1}=0.85, \sigma _{\omega 1}=0.5, \sigma _{k 2}=1.0, \sigma _{\omega 2}=0.856 \\ \beta _1=0.075, \beta _2=0.828, \beta ^*=0.09, \kappa =0.41, a_1=0.31 \end{array}\right. \end{aligned}$$

During the simulation, the thermal mass transport model is optimized by using the strain rate *S* instead of vorticity in the definition of the vortex viscosity. Additionally, a factor of 10 is used in the yield limiter instead of the 20 proposed by Menter FR in the parameter settings. These optimizations increase the computational efficiency of the Fluent software iterations^21^.

### Periodic initial boundary conditions in the study areas

The thermal performance of buildings is greatly affected by the prevailing climatic conditions. Ningxia is a region situated in the deep inland of northwest China, at the intersection of the Loess and Mongolian Plateaus. Ningxia has a temperate continental climate, which is distinguished by a significant temperature gap between day and night and moderate rainfall. The summer is usually hot and dry, while winters are cold and damp. The region receives most of its rainfall during the summer and autumn seasons, with lesser precipitation during winter.

**Figure 2:**
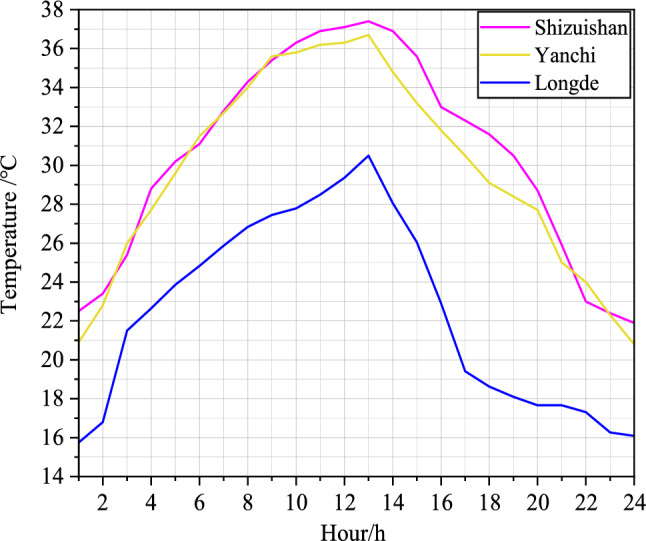
Temperature distribution of a day in the study area in summer.

The Ningxia region in China is divided into three climate zones: the middle temperate semi-humid zone, the middle temperate semi-arid zone, and the middle temperate arid zone. The current paper focuses on Shizuishan City, located in the north of Ningxia Diversion Irrigation Zone, as a typical area of the Middle Temperate Arid Zone. Yanchi County, located in the north of Yuanzhou District of Guyuan, as a representative area of the Middle Temperate Semi-Arid Zone. Similarly, Longde County, located in the south of Guyuan City, which is a typical area of the Middle Temperate Semi-Humid Zone. The study analyzes three boundary conditions of periodic temperature in the Ningxia region. This paper utilizes temperature data from a single summer day in Shizuishan, Tongxin, and Longde to establish three boundary conditions of periodic temperature for various roof structures, as illustrated in Fig. 2.

Since the temperature variation outside the farmhouse in summer was used as the initial boundary condition for the simulation, the temperature varies periodically, i.e^22,23^:

6 $$\begin{aligned} T_f=\bar{T}_f+A_f \cos \left( \frac{2 \pi t}{\tau }-\varphi \right) \end{aligned}$$

In order to simulate indoor temperature field, the outdoor temperature data in study areas was fitted to a periodic boundary cosine function as shown in Table 2. The fitting was programmed and processed as a UDF custom function to establish the initial temperature boundary conditions for doors, windows, walls, roofs, and foundations of farm buildings under natural convection conditions.

**Table 2:** Study areas to initial boundary conditions of periodic temperature cosine function.

The middle temperate arid zone	The middle temperate semi-arid zone	The middle temperate semi-humid zone
$T_1=28.65-0.554 \cos \left( -\frac{2 \pi t}{0.2873}\right)$	$T_2=29.65-0.074 \cos \left( -\frac{2 \pi t}{0.4103}\right)$	$T_3=23.25+1.873 \cos \left( -\frac{2 \pi t}{0.04352}\right)$

### Solution

**Figure 3:**
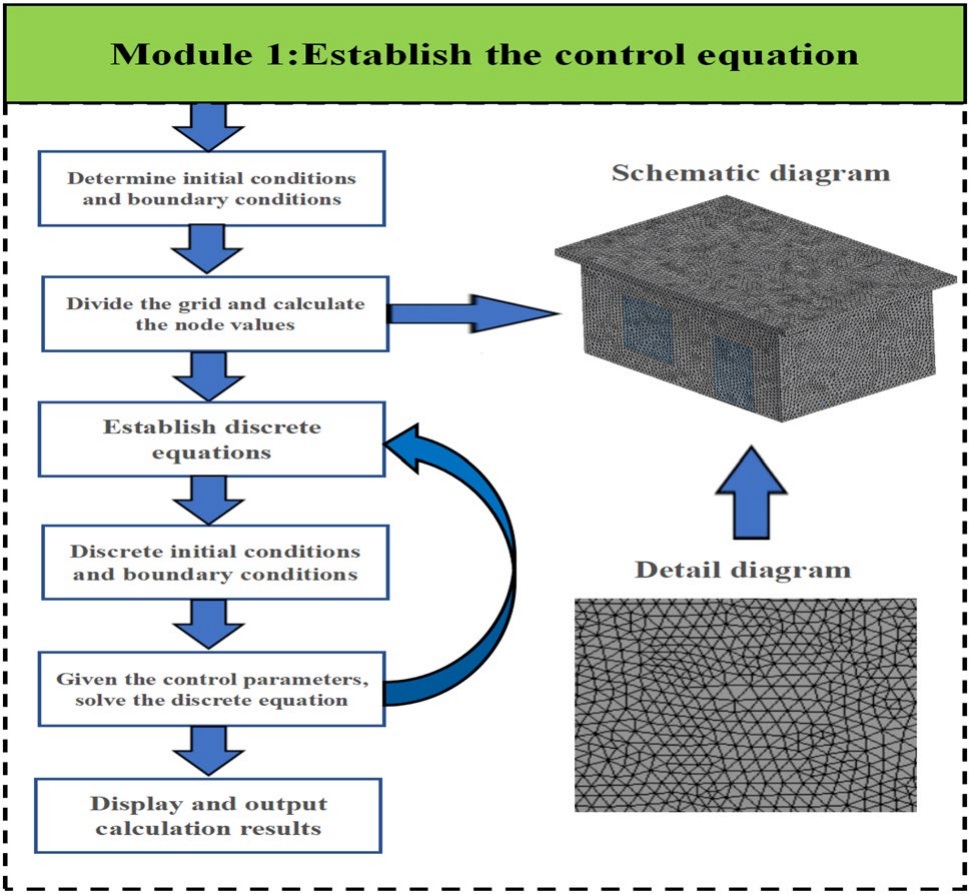
Framework diagram of the simulation process.

To meet the high accuracy requirements of numerical simulation, we established unstructured grids sized 0.1$\text{m}$ with a total of 37,000 to 42,000 nodes for the control volume by the finite element method (FEM). Additionally, we ensured that the average value of the surface mesh mass skewness was less than 0.7. The mathematical model of thermal mass transport is solved numerically by the finite element method, in which the mathematical model includes the Energy equation and the *SST* $k-\omega$ model. The *SST* $k-\omega$ model is subdivided into two equations, in which the turbulent kinetic energy *k* and turbulent frequency $\omega$ are determined by solving two independent transport equations. After the model is established, the exterior of the enclosure is determined as the solid boundary condition, and the inlet and outlet temperatures and velocities are also specified. In this academic paper, the authors utilize the SIMPLE algorithm to perform iterative calculations utilizing the simulation framework depicted in Fig. 3. The convergence of the *SST* $k-\omega$ model is judged based on 0.001, and the convergence of the Energy equation is based on 0.001.

### Verification of simulation models

**Figure 4:**
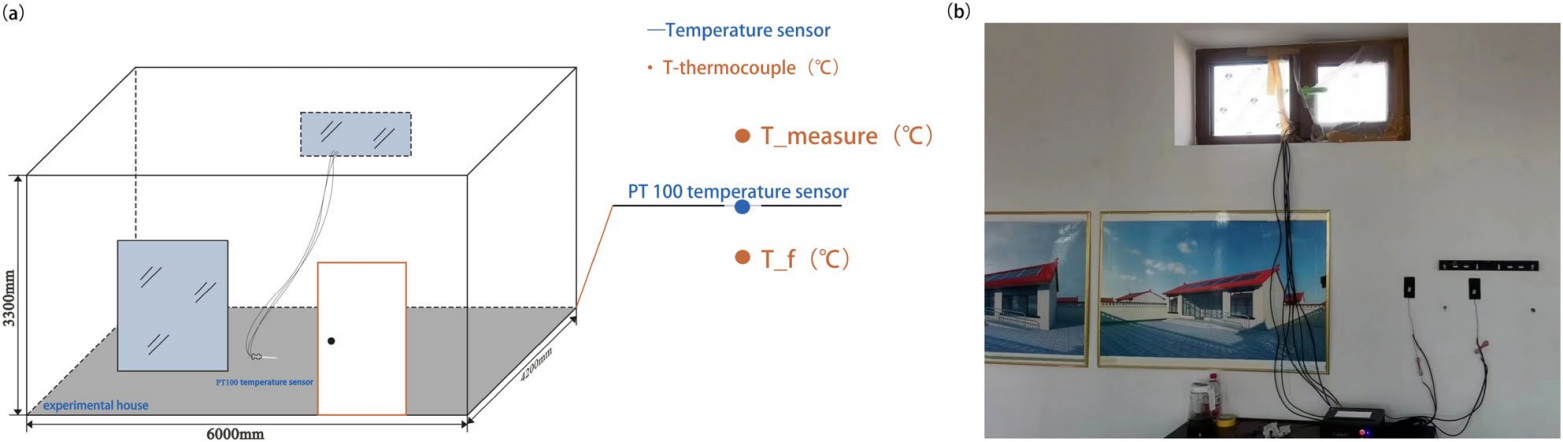
(**a**) The experimental system of model and (**b**) the arrangement diagram of the measurement equipment.

In order to verify the feasibility of thermal mass transport mathematics in dynamic simulation analysis of indoor thermal environment, an indoor temperature monitoring test was conducted in Yanchi County, Ningxia. As it depicted in Fig. 4, pt100 temperature sensor were used to measure air temperatures in the experimental house, respectively. The heat flow meter was employed to measure surface heat flows in the studied room^24^. In this experiment, the heat flow meters with the relative error lower than 5$\%$ were selected and all measurement data were recorded by PT100 Thermal Platinum Resistance Temperature Sensors. Measurement parameters of the High-precision PT100 Thermal Platinum Resistance Temperature Sensor were shown in Table 3. In order to carry out indoor thermal comfort studies, the air temperature at a vertical distance of 1.2$\text{m}$ from the floor in the human sitting condition was performed. It was used to monitore indoor temperatue for 24 hours^25^.

**Table 3:** High-precision PT100 thermalplatinum resistance temperature sensor.

The specific parameters of the equipment	
Measurement range	$-50 \sim 200 ^{\circ }$C
Measurement accuracy	Level AA
Probe size	$2 \times 5 \text{mm}$
Manufacturer	Yongyang New Energy Technology Company

**Table 4:** Comparison table of measured temperature and theoretical calculation temperature.

Hour/ $\text{h}$	1	2	3	4	5	6	7	8
Indoor temperature								
$T_{measure}$/^∘^C	20.94	21.56	22.49	22.62	22.92	23.27	23.65	23.71
$T_{simulated}$/^∘^C	21.94	22.36	22.89	22.92	23.12	23.57	23.95	24.31
Relative error	0.048	0.037	0.018	0.013	0.009	0.013	0.013	0.025

Hour/ $\text{h}$	9	10	11	12	13	14	15	16
Indoor temperature								
$T_{measure}$/^∘^C	24.22	25.49	26.04	26.32	25.5	25.65	25.17	24.38
$T_{simulate}$/^∘^C	24.72	25.69	26.44	26.92	26.1	25.95	25.37	24.58
Relative error	0.021	0.008	0.015	0.023	0.024	0.012	0.007	0.008

Hour/ $\text{h}$	17	18	19	20	21	22	23	24
Indoor temperature								
$T_{measure}$/^∘^C	24.25	23.87	23.69	23.08	22.5	22.06	21.36	21.15
$T_{simulate}$/^∘^C	24.65	23.97	23.79	23.38	22.8	22.26	21.86	21.95
Relative error	0.016	0.037	0.004	0.013	0.013	0.009	0.023	0.037

**Figure 5:**
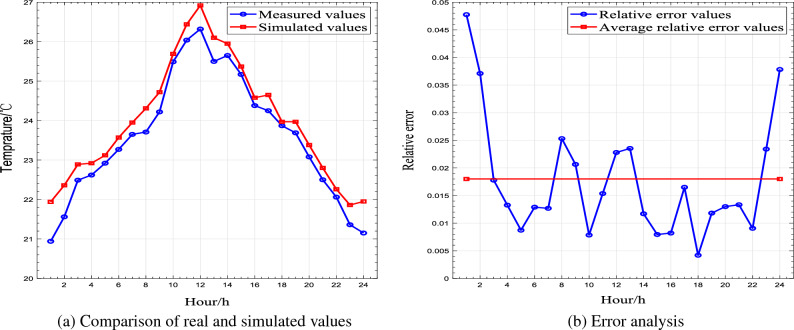
Schematics of comparison details.

The results of the simulations which based on periodic boundary conditions were shown in Table 4, tending to be consistent with the diurnal transient variations of the measured indoor air temperature of the Flat roof experimental house in Yanchi County. Values and fluctuation phases were close to each other. The relative errors between the measured temperature and simulated temperature in a period are less than 2.9^∘^C. Average relative error is 0.018. Black Ball Temperature Recorder was used to calibrate the PT 100 Temperature Sensor in this experiment, so that the monitoring data from the device can be used as experimental comparison data. The comparison between the measured temperature and simulated temperature was shown in Fig. 5. It also indicated that the mathematical model of thermal mass transport was feasible to simulate the indoor thermal environment of the farmhouse. Therefore, the mathematical physical model established by Fluent software can be used to study the periodic change rule of indoor temperature in the structural form of rural buildings.

## Results and discussion

The temperature of indoor environments in farmhouse buildings is closely related to the shape of the building. The thermal insulation performance of the building is significantly impacted by different roof structures^26^. Additionally, external temperature conditions, wind speed, wind direction, and roof forms of the house can also affect the temperature distribution inside the farmhouse. A well-designed building structure can reduce solar radiation and increase natural ventilation in summer, leading to lower indoor temperatures and energy consumption. This paper explored the impact of different roof forms on indoor temperature and energy consumption in three climate zones in Ningxia. Specifically, we simulated the indoor temperature field of three different roof forms of farmhouse structures and extracted the central section of the computational fluid domain along the z-axis for analysis. For example, central section of the Flat roof had a height of 4.2$\text{m}$ and a width of 4.2$\text{m}$, central section of the Equal-sloped roof had a height of 4.270$\text{m}$ and a width of 4.2$\text{m}$, and central section of the Non-equal-sloped roof had a height of 4.289$\text{m}$ and a width of 4.2$\text{m}$. Finally, we observed indoor temperature distribution longitudinally by intercepting 1.2$\text{m}$ surfaces.

The selection of wall materials in this simulation is based on the farmhouse structures in the field study area. The EPS solid clay brick wall (External Wall 1) was used for the farmhouse structure in Shizuishan City, while the combined adobe brick and clay brick wall (External Wall 2) was used in Yanchi County, and the solid clay brick wall (External Wall 3) was used in Longde County. The heat transfer coefficients and thicknesses of these wall materials vary, leading different numerical simulation outcomes^27,28^. Combined with the construction technology of farmhouse walls in the three study areas, we selected the above composite wall as the solid boundary for the simulations.

### Experimental house of the middle temperate arid zone

#### Comparison of temperature distribution at the center of experimental house in Shizuishan city

In this paper, the outdoor air temperature in Shizuishan City was used as the initial condition of the experimental house in the Middle Temperate Arid Zone, and the temperature distribution in the test house at the center of different roof forms was simulated after 24 hours according to the boundary conditions of $T_{1}$^29^.

**Figure 6:**
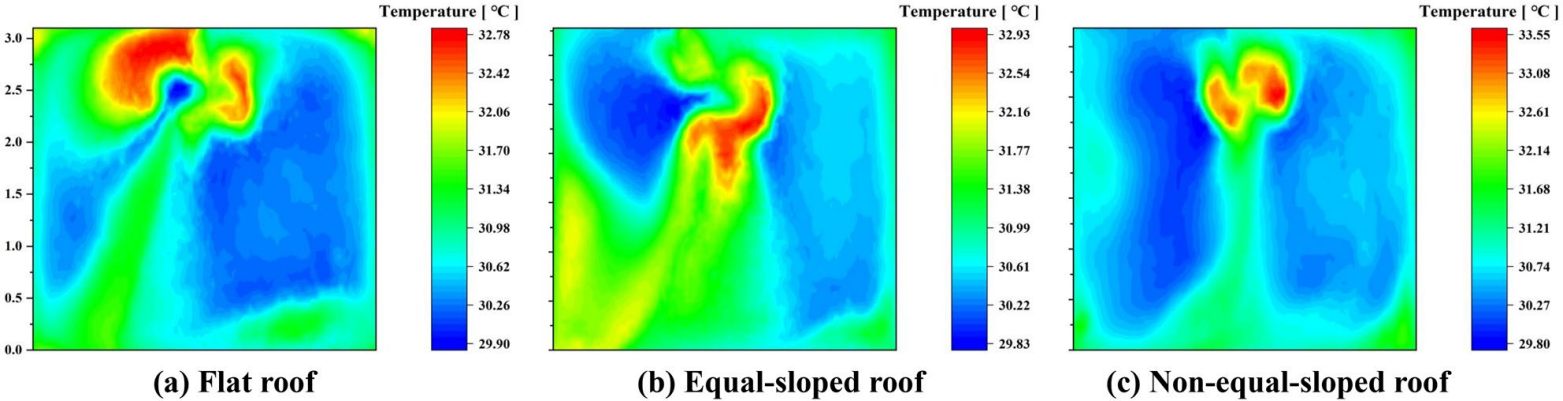
Temperature simulations of summer indoor center locations of different roofs of farmhouse structures in the Middle Temperate Arid Zone of Ningxia.

Under 24-hour periodic thermal action $T_{1}$ conditions, the cross-sectional cloud maps of a single test house in the Middle Temperate Arid Zone in the direction of the airflow vector normal were shown in Fig. 6, and the location of the cross-section was in the center of the experimental house.

**Table 5:** Comparison table of temperature distribution at the center section of experimental house in Shizuishan city.

Experimental house	Average temperature (^∘^C)	Highest temperature (^∘^C)	Distribution of the highest temperature ($\text{m}$)	Lowest temperature (^∘^C)	Distribution of the lowest temperature ($\text{m}$)	Temperature difference ($\text{m}$)
Flat roof	30.85	32.78	$2.25 \sim 3.25$	29.91	$0.5 \sim 3$	2.87
Equal-sloped roof	30.99	32.92	$1.5 \sim 2.75$	29.84	$1.5 \sim 2.5$	3.08
Non-equal-sloped roof	30.75	33.54	$2.25 \sim 2.75$	29.81	$0.25 \sim 2.25$	3.73

As shown in Table 5 (Supplementary Information 1), the average temperature of the experimental room with Flat roof in the section was 30.85 ^∘^C; the highest temperature of the section was 32.78 ^∘^C, which was distributed in the section from 2.25 to 3.25$\text{m}$; the lowest temperature of the section was 29.91 ^∘^C, which was distributed in the section from 0.5 to 3$\text{m}$. The temperature difference of the section was 2.87 ^∘^C, because the door and window cavity were open during the simulation^30^. The outdoor temperature was 22.4 ^∘^C currently, which caused the section temperature in the normal direction of the doorway reached the minimum, indicating that the temperature in the normal direction of the airflow vector of the doorway was significantly influenced by the periodic thermal effect. The simulation results showed that the average air temperature at the height of 1.2 $\text{m}$ in the human activity area is at 30.58 ^∘^C.

The average temperature of the experimental room with Equal-sloped roof in the section was 30.99 ^∘^C. The highest temperature of the section was 32.92 ^∘^C, distributed in the section from 1.5 to 2.75$\text{m}$, which was 0.14 ^∘^C higher than the highest temperature of the section with Flat roof. The lowest temperature of the section was 29.84 ^∘^C, distributed in the section from 1.5 to 2.5m. The temperature difference of the section was 3.08 ^∘^C, and the minimum temperature of this section was significantly influenced by the periodic thermal effect in the direction of the window cavity airflow, making the low temperature region mainly concentrated in the window cavity region. The average air temperature at the height of 1.2 $\text{m}$ in the human activity area was 30.86 ^∘^C, which was 0.28 ^∘^C higher than that of Flat roof.

The average temperature of the experimental room with Non-equal-sloped roof in the section was 30.75 ^∘^C, which was the lowest average temperature of all roof forms; the highest temperature of the section was 33.54 ^∘^C, distributed in the section from 2.25 to 2.75$\text{m}$, and the comparison of the highest temperature of the section in different roof constructions revealed that: Non-equal-sloped roof was greater than Equal-sloped roof while Equal-sloped roof was greater than Flat roof. The lowest temperature of the section was 29.81 ^∘^C, distributed in the section from 0.25 to 2.25$\text{m}$; the temperature difference of the section was 3.73 ^∘^C. Among the three roof forms, the temperature difference of Non-equal-sloped roof was the largest, which indicated that the cooling effect of Non-equal-sloped roof was obvious. At this time, the average air temperature at the height of 1.2 $\text{m}$ in the human activity area was 30.64 ^∘^C, which was found under the comparison of three roof construction conditions: Equal-sloped roof was greater than Non-equal-sloped roof while Non-equal-sloped roof was greater than Flat roof.

#### Comparison of phase delay angle and delay time of the experimental house in Shizuishan city

From the results of numerical simulation, we found that the cyclic thermal action had differences on the distribution of indoor temperature field for different roof structures. At the same time, the roles of roof forms applicable to farm buildings on different temperate zones in summer thermal insulation were also different^31^.

The comparison between the simulation results of indoor temperature field in the Middle Temperate Arid Zone of different roof forms in Shizuishan City under the same parameter condition settings was shown as follows^32^:

**Figure 7:**
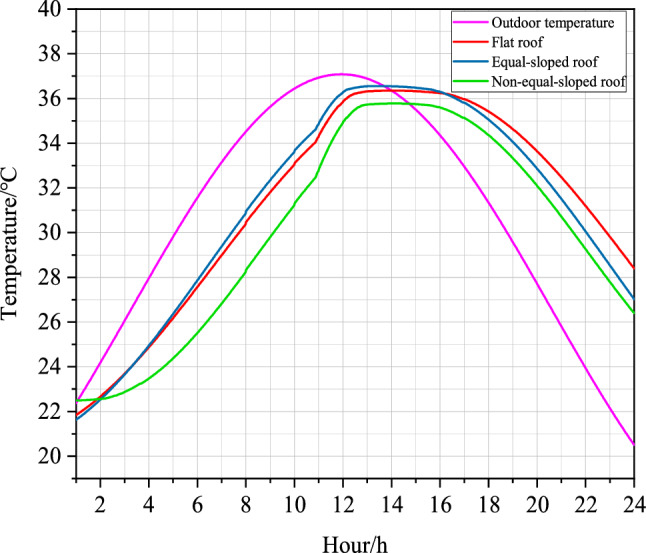
Folding line diagram of summer indoor and outdoor temperatures of different roofs of farmhouse structures in the Middle Temperate Arid Zone of Ningxia.

As shown in Fig. 7, the amplitude of outdoor temperature in Shizuishan City, the Middle Temperate Arid Zone in Ningxia, was 5.55 ^∘^C. The amplitude of Flat roof temperature was 4.52 ^∘^C, the amplitude of Equal-sloped roof was 4.76 ^∘^C, and the amplitude of Non-equal-sloped roof was 5.23 ^∘^C. Since in the thermal theory of the building, the total attenuation degree $v_{0}$ of the wall is defined as the ratio of the wall temperature $A_{if}$ for different roof forms caused by the periodic thermal action of the outdoor temperature to the outdoor temperature amplitude $A_{e}$, i.e.

7 $$\begin{aligned} v_{0}=\frac{A_{if}}{A_{e}}. \end{aligned}$$

Therefore, the comparison of the attenuation degree of temperature fluctuation from outdoor space to wall space for different roofing structures was found to be: Non-equal-sloped roof was greater than Equal-sloped roof and Equal-sloped roof was greater than Flat roof^33^.

As shown in Fig. 7, the maximum outdoor temperature in Shizuishan City, the Middle Temperate Arid Zone in Ningxia, occurred at 11:56 $\text{am}$. The maximum indoor temperature in Flat roofs appeared at 14:04 $\text{am}$, the maximum indoor temperature in Equal-sloped roofs occurred at 13:29 $\text{am}$, and the maximum indoor temperature in Non-equal-sloped roofs occurs at 14:07 am. In the theory of building thermal engineering, the moment defining the appearance of the maximum value of the external temperature is usually defined as $\tau _{e,max}$, and the moment defining the appearance of the maximum temperature of the inner surface of the walls of different roof forms as $\tau _{if,max}$. The difference between them is called the total delay time of the temperature wave as it passes through the wall, and let’s denote it by $\xi _{0}$^34^. Therefore, the total delay time for the temperature wave to pass through the Flat roof structure was 2.13$\text{h}$, the total delay time for the temperature wave to pass through the Equal-sloped roof structure was 1.55$\text{h}$, and the total delay time for the temperature wave to pass through the Non-equal-sloped roof structure was 2.18$\text{h}$.

$\xi _{0}$ has the following relationship with the total phase delay angle $\phi _{0}$:

8 $$\begin{aligned} \phi _{0}=\frac{360\xi _{0}}{Z}. \end{aligned}$$

where *Z* indicates the period of temperature fluctuation, $\text{h}$. Therefore, the phase delay angle of temperature fluctuation of Flat roof structure was 31.95^∘^, the phase delay angle of temperature fluctuation of Equal-sloped roof structure was 23.25^∘^, and the phase delay angle of temperature fluctuation of Non-equal-sloped roof structure was 32.7^∘^. As discussed, this fact demonstrates that the phase delay angle of temperature fluctuation of different roof structures from outdoor space to wall space was found as follows: Non-equal-sloped roof is greater than Flat roof and Flat roof was greater than Equal-sloped roof.

### Experimental house of the middle temperate semi-arid zone

#### Comparison of temperature distribution at the center of experimental house in Yanchi county

In this paper, the outdoor air temperature in Yanchi County was used as the initial condition of the experimental room in the Middle Temperate Semi-Arid Zone, and the temperature distribution in the experimental room at the center of different roof forms was simulated after 24 hours according to the boundary conditions of $T_{2}$.

**Figure 8:**
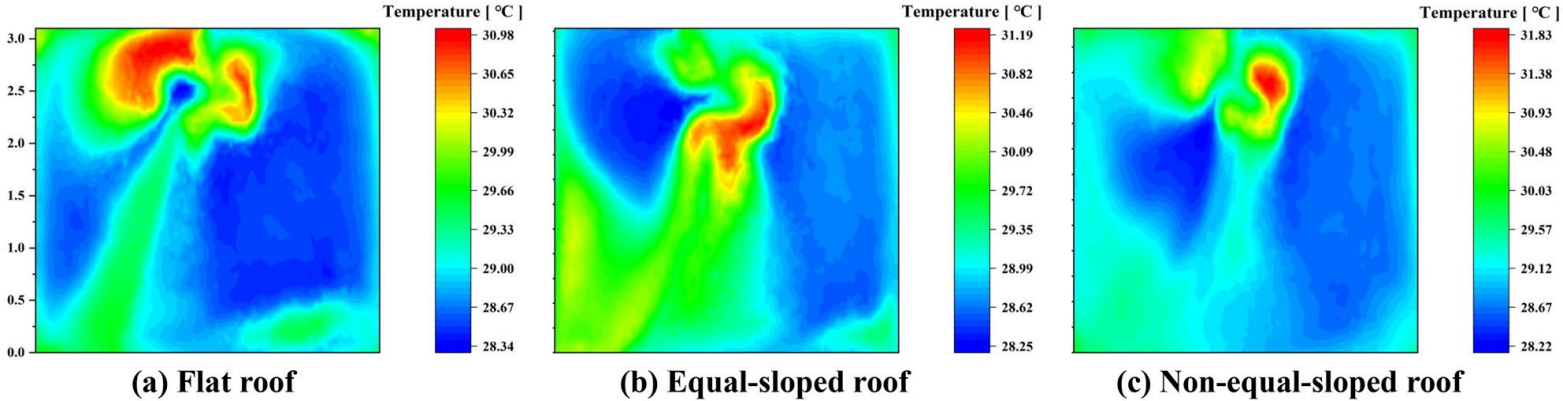
Temperature simulations of summer indoor center locations of different roofs of farmhouse structures in the middle temperate semi-arid zone of Ningxia.

Under 24-hour periodic thermal action $T_{2}$ conditions, the cross-sectional cloud maps of a single test house in the Middle Temperate Semi-Arid Zone in the direction of the airflow vector normal were shown in Fig. 8, and the location of the cross-section was located in the center of the experimental house.

**Table 6:** Comparison table of temperature distribution at the center section of experimental house in Yanchi county.

Experimental house	Average temperature (^∘^C)	Highest temperature (^∘^C)	Distribution of the highest temperature ($\text{m}$)	Lowest temperature (^∘^C)	Distribution of the lowest temperature ($\text{m}$)	Temperature difference ($\text{m}$)
Flat roof	29.07	30.97	$2.1 \sim 3.2$	28.35	$0.25 \sim 2.5$	2.62
Equal-sloped roof	29.20	31.19	$1.5 \sim 2.7$	28.26	$1.5 \sim 2.5$	2.93
Non-equal-sloped roof	29.10	31.82	$2.25 \sim 2.75$	28.22	$1.0 \sim 2.0$	3.60

As shown in Table 6 (Supplementary Information 2), the average temperature of experimental room with Flat roof in the section was 29.07 ^∘^C. The highest temperature of the section is 30.97 ^∘^C, which was distributed from 2.1 to 3.2$\text{m}$ in this section. The lowest temperature in the section was 28.35 ^∘^C, which was distributed in the section from 0.25 to 2.5$\text{m}$. The temperature difference of the section was 2.62 ^∘^C, since the door and window cavity were open during the simulation. The outdoor temperature was 22.8 ^∘^C at this time, resulting in the lowest section temperature at the normal direction of the door opening. This phenomenon indicated that the temperature in the normal direction of the airflow vector of the door opening was significantly influenced by the periodic thermal effect. The simulation results showed that the average air temperature at the height of 1.2 $\text{m}$ in the human activity area was 28.80 ^∘^C.

The average temperature of experimental room with Equal-sloped roof in the section was 29.20 ^∘^C, and the highest temperature of the section was 31.19 ^∘^C, distributed in the section from 1.5 to 2.7$\text{m}$, which was 0.22 ^∘^C higher than the highest temperature of Flat roof section. The lowest temperature of the section was 28.26 ^∘^C, which was distributed in the section from 1.5 to 2.5$\text{m}$. The temperature difference of the section was 2.93 ^∘^C. The minimum temperature of this section was still influenced by the periodic thermal action in the direction of the airflow of the door and window openings, distributed in the location of the door and window openings in the section. The average air temperature at the height of 1.2 $\text{m}$ in the human activity area was 29.08 ^∘^C, which was 0.28 ^∘^C higher than that of Flat roof.

The average temperature of the experimental room with Non-equal-sloped roof in the section was 29.10 ^∘^C, and the highest temperature of the section is 31.82 ^∘^C, which was distributed in the section from 2.25 to 2.75$\text{m}$. The comparison of the highest temperature of the section in different roof structures was that: Non-equal-sloped roof was greater than Equal-sloped roof while Equal-sloped roof was greater than Flat roof. The lowest temperature of the section was 28.22 ^∘^C, which was distributed in the section from 1.0 to 2.0$\text{m}$. The temperature difference of the section was 3.6 ^∘^C. The maximum temperature difference of such roof form indicated that the cooling effect of Non-equal-sloped roof was obvious. At this time, the average air temperature at 1.2m height of human activity area was 28.81 ^∘^C, and this temperature was found in the comparison of three roof construction conditions: Non-equal- sloped roof was greater than Flat roof while Flat roof was greater than Equal-sloped roof.

#### Comparison of phase delay angle and delay time of the experimental house in Yanchi county

The comparison between the simulation results of indoor temperature field in the Middle Temperate Semi-Arid Zone of different roof forms in Yanchi County under the same parameter condition settings was shown as follows:

**Figure 9:**
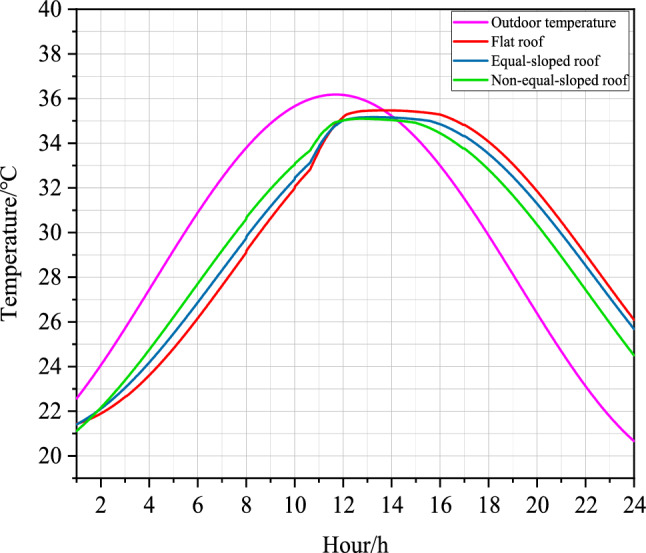
Folding line diagram of summer indoor and outdoor temperatures of different roofs of farmhouse structures in the middle temperate semi-arid zone of Ningxia.

As it depicted in Fig. 9, the amplitude of outdoor temperature in Yanchi County, the Middle Temperate Semi-Arid Zone of Ningxia, was 5.51 ^∘^C. The amplitude of Flat roof temperature was 4.89 ^∘^C, the amplitude of Equal-sloped roof was 4.60 ^∘^C, and the amplitude of Non-equal-sloped roof was 4.57 ^∘^C. Therefore, the comparison of the attenuation of temperature fluctuation from outdoor space to wall space for different roof structures was found to be: Non-equal-sloped roof was greater than Equal-sloped roof and Equal-sloped was greater than Flat roof.

As shown in Fig. 9, the maximum outdoor temperature in Yanchi County, the Middle Temperate Semi-Arid Zone of Ningxia, appeared at 11:41 $\text{am}$. The maximum indoor temperature in Flat roof appeared at 13:41 $\text{am}$, the maximum indoor temperature in Equal-sloped roof appeared at 13:16 $\text{am}$, and the maximum indoor temperature in Non-equal-sloped roof appeared at 13:43 $\text{am}$. Therefore, the total delay time when the temperature wave passed through the Flat roof structure was 2$\text{h}$, the total delay time when the temperature wave passed through the Equal-sloped roof structure was 1.59$\text{h}$, and the total delay time when the temperature wave passed through the Non-equal-sloped roof structure was 2.03$\text{h}$.

Then, according to the calculation of Eq. (7), the phase delay angle of temperature fluctuation of Flat roof structure is 30^∘^, the phase delay angle of temperature fluctuation of Equal-sloped roof structure was 23.85^∘^, and the phase delay angle of temperature fluctuation of Non-equal-sloped roof structure was 30.45^∘^. As discussed, this fact demonstrated that the phase delay angle of temperature fluctuation of different roof structures from outdoor space to wall space was found to be: Non-equal-sloped roof was greater than Flat roof and Flat roof was greater than Equal-sloped roof.

### Experimental house of the middle temperate semi-humid zone

#### Comparison of temperature distribution at the center of experimental house in Longde county

In this paper, the outdoor air temperature in Longde County was used as the initial condition of the experimental house in the Middle Temperate Semi-humid Zone, and the temperature distribution in the test room at the central location under different roof forms was simulated after 24 hours according to the boundary conditions of $T_{3}$.

**Figure 10:**
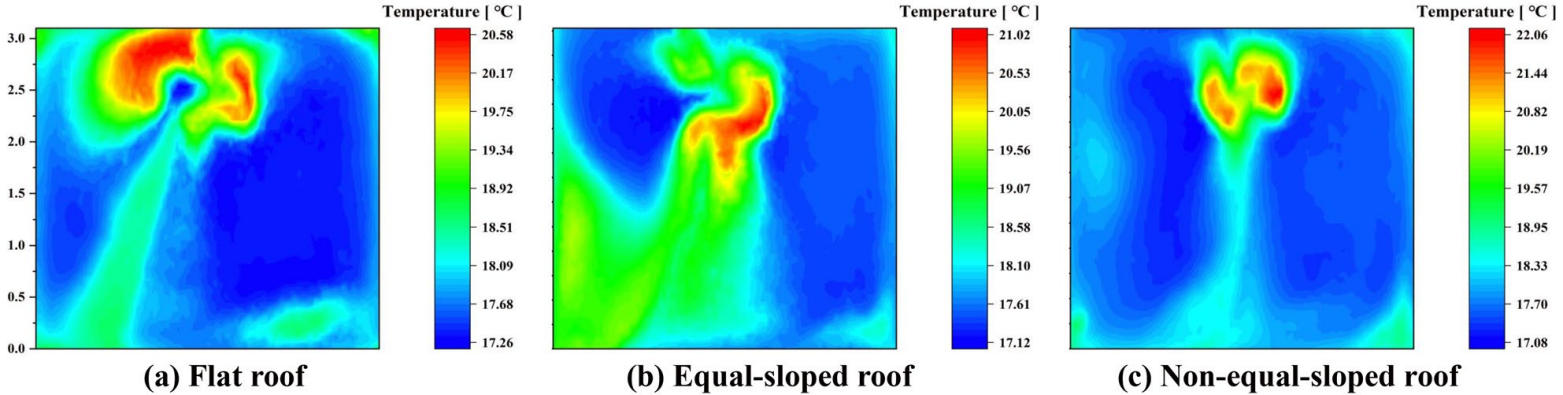
Temperature simulations of summer indoor center locations of different roofs of farmhouse structures in the middle temperate semi-humid zone of Ningxia.

Under 24-hour periodic thermal action conditions of $T_{3}$, the cross-section cloud maps of a single test house in the Middle Temperate Semi-Humid Zone in the direction of the airflow vector normal were shown in Fig. 10. The location of the cross-section was located at the center of the experimental house.

**Table 7:** Comparison table of temperature distribution at the center section of experimental house in Longde county.

Experimental house	Average temperature (^∘^C)	Highest temperature (^∘^C)	Distribution of the highest temperature ($\text{m}$)	Lowest temperature (^∘^C)	Distribution of the lowest temperature ($\text{m}$)	Temperature difference ($\text{m}$)
Flat roof	18.04	20.57	$2.25 \sim 3.1$	17.27	$0.5 \sim 2.0$	3.30
Equal-sloped roof	18.20	21.02	$1.5 \sim 2.5$	17.13	$0.5 \sim 1.5$	3.89
Non-equal-sloped roof	17.92	22.06	$2.0 \sim 3.0$	17.08	$0.5 \sim 2.5$	4.98

As shown in Table 7 (Supplementary Information 3), the average temperature of experimental room with Flat roof in the section was 18.04^∘^C. The highest temperature of the section was 20.57^∘^C, which was distributed in the section from 2.25 to 3.1$\text{m}$. The lowest temperature of the section was 17.27^∘^C, which was distributed in the section from 0.5 to 2$\text{m}$. The temperature difference of the section was 3.3 ^∘^C, because the door and window cavity were open during the simulation state. The outdoor temperature was 15.7 ^∘^C at this time, causing the section temperature at the direction normal to the doorway reached the lowest, indicating that the temperature in the normal direction of the airflow vector of the doorway was significantly influenced by the periodic thermal effect. The simulation results showed that the average air temperature at the height of 1.2 $\text{m}$ in the human activity area was 17.68 ^∘^C.

The average temperature of the experimental room with Equal-sloped roof in the section was 18.20 ^∘^C. The highest temperature of the section was 21.02 ^∘^C, distributed in the section from 1.5 to 2.5m, which was 0.43 ^∘^C higher than the maximum temperature of the section with Flat roof. The lowest temperature of the section was 17.13 ^∘^C, which was distributed in the section from 0.5 to 1.5$\text{m}$. The temperature difference of the section was 3.89 ^∘^C, and the minimum temperature of the section was still influenced by the periodic thermal action in the direction of the airflow in the doorway. The average air temperature at the height of 1.2m in the human activity area was 18.06 ^∘^C, which was 0.38 ^∘^C higher than that of the Flat roof.

The average temperature of the experimental room with Non-equal-sloped roof in the sectionwas 17.92 ^∘^C. The highest temperature of the section was 22.06 ^∘^C, distributed in the section from 2.0 to 3.0$\text{m}$, and the highest temperature of the section in different roof structures was found: Non-equal-sloped roof was greater than Equal-sloped roof and the Equal-sloped roof was greater than Flat roof. The lowest temperature of the section was 17.08 ^∘^C, distributed in the section from 0.5 to 2.5$\text{m}$. The temperature difference of the section was 4.98 ^∘^C, the distribution range of the lowest temperature of this section increased sequentially compared with Flat roof and Equal-sloped roof, which indicated that the influence of the heat flow of the doorway on its temperature distribution gradually decreases, and the average air temperature at the height of 1.2 $\text{m}$ in the human activity area was 17.15 ^∘^C at this time, which was the lowest temperature under the three roof construction conditions.

#### Comparison of phase delay angle and delay time of the experimental house in Longde county

The comparison between the simulation results of indoor temperature field in the Middle Temperate Semi-humid Zone of different roof forms in Longde County under the same parameter condition settings was shown as follows:

**Figure 11:**
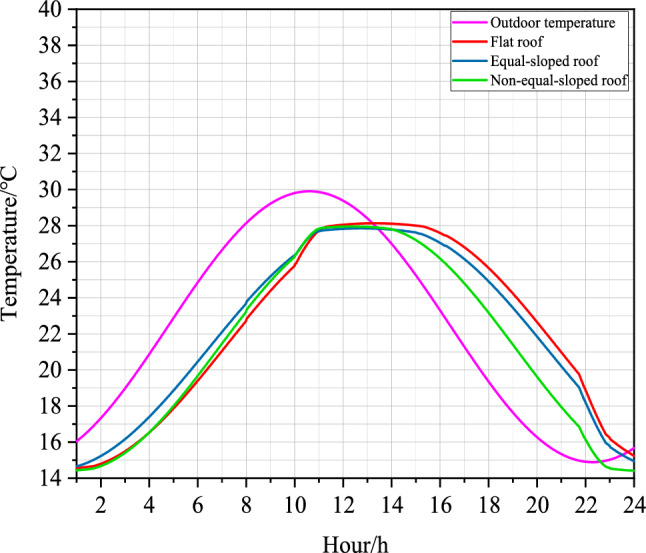
Folding line diagram of summer indoor and outdoor temperatures of different roofs of farmhouse structures in the middle temperate semi-humid zone of Ningxia.

As it depicted in Fig. 11, the amplitude of outdoor temperature in Longde County, the Middle Temperate Semi-Humid Zone of Ningxia, was 7.41 ^∘^C. The amplitude of Flat roof temperature was 5.69 ^∘^C, the amplitude of Equal-sloped roof was 5.40 ^∘^C, and the amplitude of Non-equal-sloped roof was 6.35 ^∘^C. Therefore, the comparison of the attenuation of temperature fluctuation from outdoor space to wall space for different roof structures was found to be: Non-equal-sloped roof was greater than Flat roof and Flat roof was greater than Equal-sloped roof.

As shown in Fig. 11, the maximum outdoor temperature in Longde County, the Middle Temperate Semi-Humid Zone of Ningxia, appeared at 10:36 $\text{am}$. The maximum indoor temperature in Flat roof appeared at 13:19 $\text{am}$, the maximum indoor temperature in Equal-sloped-roof appeared at 12:44 $\text{am}$, and the maximum indoor temperature in Non-equal-sloped roof appeared at 12:22 $\text{am}$. Therefore, the total delay time was 2.73 $\text{h}$ when the temperature wave passed through Flat roof structure. when the temperature wave passes through Equal-sloped roof structure, the total delay time was 2.14 $\text{h}$. When the temperature wave passes through Non-equal-sloped roof structure, the total delay time was 2.78 $\text{h}$.

According to the calculation of Eq. (7), the phase delay angle of temperature fluctuation of Flat roof structure was 40.95^∘^, the phase delay angle of temperature fluctuation of Equal-sloped roof structure was 32.1^∘^, and the phase delay angle of temperature fluctuation of Non-equal-sloped roof structure was 41.7^∘^, which indicated that the phase delay angle of temperature fluctuation of different roof structures from outdoor space to wall space is found as follows: Non-equal-sloped roof was greater than Flat roof and Flat roof was greater than Equal-sloped roof .

## Conclusions

This paper analyzes the heat transfer of traditional farm buildings in Ningxia Autonomous Region in different climate zones. We established a mathematical model of thermal mass transfer in a single experimental room and comparatively analyzed the effects of different roof forms on indoor temperature distribution in summer in Ningxia. The following conclusions were drawn:

- All kinds of roof form, the indoor thermal environment was significantly affected after 24 hours of periodic thermal interaction. We have synthesized and characterized the indoor temperature that the direction normal to the airflow vector of the door and window holes, specifically in the direction of the rear window of the indoor centre section in summer. Kinetic analyses suggest that there is a need to enhance the summer thermal insulation measures for doors and windows of Ningxia farmhouse buildings.
- Based on results of the numerical simulations, the maximum indoor temperatures in all the experimental houses with Flat roofs in the climatic zone are lower than in the other roof types. The roof form in which the lowest value of the average air temperature at a height of 1.2$\text{m}$ in the human activity area of the experimental house within the Middle Temperate Arid Zone occurred is Flat roof; the Middle Temperate Semi-Arid Zone is the Equal-sloped roof; and the Middle Temperate Semi-humid Zone is Non-equal-sloped roof.
- By way of calculating and comparing the degree of attenuation of temperature fluctuations in the experimental room under different roof forms, we have drawn some conclusions. The maximum indoor temperature attenuation of the experimental house located in the Middle Temperate Arid Zone is Non-equal-sloped roof. While the Flat roof had the largest attenuation degree in the Middle Temperate Semi-arid Zone, and the Non-equal-sloped roof had the largest attenuation degree in the Middle Temperate Semi-humid Zone. Moreover, the temperature fluctuation on the three types of climate zones shows Non-equal-sloped roof attenuates the most temperature values.
- The Non-equal-sloped roof of farmhouse structures in Ningxia region showed the best performance in terms of indoor temperature attenuation degree and phase delay in different temperature zones.

### Supplementary Information

Supplementary Information 1.

Supplementary Information 2.

Supplementary Information 3.

## Data Availability

The data that support the findings of this study are available from the corresponding author upon reasonable request.

## List of symbols

*K*: Heat transfer coefficient, $\text{W} /\left( \mathrm {m^2} \cdot \text{K} \right)$
*d*: Thickness of wall material, $\text{mm}$
$A_{f}$: Amplitude of outdoor temperature wave on a certain day, ^∘^C
*t*: The time of recording the temperature on a given day, which according to the CMA statistics is known to be recorded once an hour on average, $\text{s}$
$\xi _{0}$: Total delay time, $\text{h}$
$$\lambda$$: Thermal conductivity, $$\text{W} /\left( \text{m} \cdot \text{K} \right)$$
$\bar{T}_f$: The average temperature of a day in summer at a place in Ningxia, ^∘^C
$\varphi$: Initial phase of temperature wave
$\tau$: The daily cycle of temperature fluctuations for a day, $\text{h}$
$\phi _{0}$: The total phase delay angle
